# Dinaciclib potently suppresses MCL-1 and selectively induces the cell death in human iPS cells without affecting the viability of cardiac tissue

**DOI:** 10.1038/srep45577

**Published:** 2017-03-31

**Authors:** Khaled Alsayegh, Katsuhisa Matsuura, Hidekazu Sekine, Tatsuya Shimizu

**Affiliations:** 1 Institute of Advanced Biomedical Engineering and Science, Tokyo Women’s Medical University, 8-1 Kawada-cho, Shinjuku, Tokyo, 162-8666, Japan; 2 King Abdullah International Medical Research Center (KAIMRC), King Saudi bin Abdulaziz University for Health Sciences, Jeddah, Saudi Arabia; 3 Department of Cardiology, Tokyo Women’s Medical University, 8-1 Kawada-cho, Shinjuku, Tokyo, 162-8666, Japan

## Abstract

Induced pluripotent stem (iPS) cells hold great potential for being a major source of cells for regenerative medicine. One major issue that hinders their advancement to clinic is the persistence of undifferentiated iPS cells in iPS-derived tissue. In this report, we show that the CDKs inhibitor, Dinaciclib, selectively eliminates iPS cells without affecting the viability of cardiac cells. We found that low nanomolar concentration of dinaciclib increased DNA damage and p53 protein levels in iPSCs. This was accompanied by negative regulation of the anti-apoptotic protein MCL-1. Gene knockdown experiments revealed that p53 downregulation only increased the threshold of dinaciclib induced apoptosis in iPS cells. Dinaciclib also inhibited the phosphorylation of Serine 2 of the C-terminal domain of RNA Polyemrase II through CDK9 inhibition. This resulted in the inhibition of transcription of *MCL-1* and the pluripotency genes, *NANOG* and *c-MYC*. Even though dinaciclib caused a slight downregulation of MCL-1 in iPS-derived cardiac cells, the viability of the cells was not significantly affected, and beating iPS-derived cardiac cell sheet could still be fabricated. These findings suggest a difference in tolerance of MCL-1 downregulation between iPSCs and iPS-derived cardiac cells which could be exploited to eliminate remaining iPS cells in bioengineered cell sheet tissues.

Cell-based regenerative medicine is currently considered as one of the most promising methods for the treatment of numerous types of damaged/defective tissues and organs. Therapies that involve the injection of cells have already been successfully performed clinically^1^,^2^,^3^. Although these studies and others have demonstrated great potential of cell therapy, loss of cells following injection may be a limiting factor that might diminish the therapeutic potential^4^,^5^,^6^. To overcome this, regenerative therapies that employ tissue-engineering technologies have been applied to patients with impaired tissue or organ function^7^,^8^,^9^. These methods have been shown to improve the engraftment and survival of transplanted cells^10^,^11^.

Human iPS cells provide a promising cell source from which numerous types of tissues may be engineered and in varying amounts. Therefore, their utilization would expand the range of clinical application of regenerative medicine. However, a number of issues remain to be resolved before their full potential can be realized in clinics. Mainly, the risk of tumor formation that could result from the presence remaining undifferentiated iPS cells in bioengineered tissue. Because around 1 × 10^9^ of iPS-derived cells are required for tissue engineering to treat heart failure, the risk of iPS contamination increases substantially^12^. This risk is compounded when using delivery techniques such as, cardiac cell-sheet transplantation, which has been shown to improve engraftment and survival of cells when compared to conventional cell injection^11^. Therefore, there is a pressing need to develop highly efficient techniques that would selectively eliminate undifferentiated iPS cells in iPS-derived bioengineered cardiac tissue.

Dinaciclib, cyclin-dependent kinases inhibitor, is currently undergoing phase I/II for advanced breast cancer and non-small cell lung carcinoma^13^,^14^,^15^. It has 50% kinase inhibitory concentrations of 1, 1, 3 and 4 nM for CDK2, CDK5, CDK1 and CDK9, respectively^16^. Recently it has been demonstrated that the inhibition of CDK1 (or its binding proteins, cyclin A2 and cyclin B1/B2) in murine embryonic stem cells (ESCs) induced apoptosis^17^. Furthermore, the treatment with the small molecule, Purvalanol A for CDK1 inhibition was found to selectively induce the cell death of mouse and human ESCs and not their differentiated derivatives. On the other hand, specific RNA interference (siRNA)-mediated CDK1 downregulation in human ES and iPS cells has been shown to cause an increase in DNA damage, an increase in differentiation and an accumulation of cells in the G2 phase of the cell cycle with no increase in apoptosis^18^. Therefore, further elucidation is required to discern the effect of CDK1 chemical inhibition on human iPS cells.

Dinaciclib also inhibits the kinase activity of CDK9, which with its binding partner, CYCLIN T (T1, 2 or 3), make the active form of the positive transcription elongation factor b (P-TEFb) complex^19^. In the P-TEFb complex, CDK9 carries out the phosphorylation of RNA polymerase II at the carboxyl-terminal domain (CTD) serine 2, which results in the elongation of nascent transcripts and the production of complete mRNAs^20^. CDK9 has been shown to bind the *Oct4* enhancer site and the promoters of *Sox2* and *Nanog* in murine ESCs and its chemical inhibition causes a significant reduction in the transcription of those genes^21^. Further elucidation is needed to examine the effect of such treatment on the viability of pluripotent stem cells.

In the present report we demonstrate that dinaciclib treatment induces cell death in human iPS cells, but not in their derived cardiac cells. This treatment may allow the fabrication of bioengineered cardiac tissue devoid of the teratoma causing iPS cells. Indeed, we have found that dinaciclib selectively eliminated iPSCs when co-cultured with normal human cardiac fibroblasts. The observed cell death in iPS cells following dinaciclib treatment correlated with an increase in p53 protein levels and a decrease in the transcription of the pluripotency genes *NANOG* and *c-MYC* and the anti-apoptotic protein, *MCL-1*. Furthermore, dinaciclib treatment induced an increase in DNA damage in iPS cells that was less pronounced in iPS-derived cardiomyocytes. Taken together, dinaciclib treatment of iPS-derived cardiac tissue might be an efficient step to purge out residual iPS cells during the process of tissue engineering for regenerative medicine.

## Results

### Low concentration dinaciclib treatment induces apoptosis in human iPS cells

**Figure 1:**
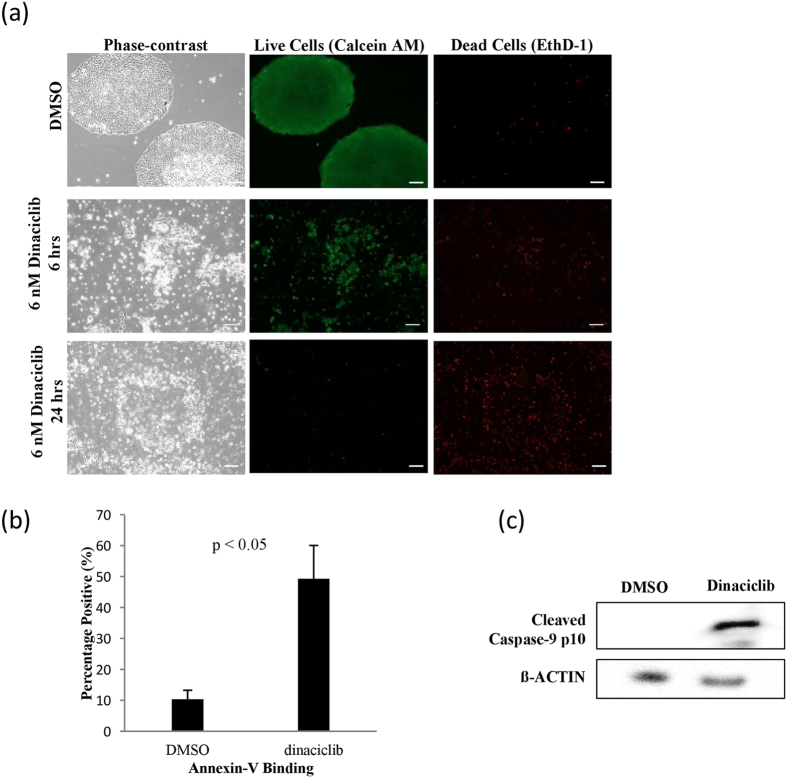
Dinaciclib induces apoptosis in feeder-free human iPS cells. (**a**) Representative images of iPS cells cultured on laminin E8 fragment treated with DMSO for 24 hrs (top panel) or 6 nM dinaciclib for 7 (middle panel) and 24 hrs (bottom panel) and then stained with Calcein AM to detect live cells (green) and Ethidium homodimer 1 (EthD-1) to detect dead cells (red). Scale bar = 100 μm (**b**) iPS cells were treated with DMSO or 6 nM dinaciclib for 6 hrs and then incubated briefly with Annexin V-FITC and analyzed for Annexin V binding using flow cytometry (n = 3). (**c**) iPS cells were treated with DMSO or 6 nM dinaciclib for 6 hrs. Cells were then harvested and whole-cell lysates were used for Western Blot analysis and detection of the active cleaved p10 subunit of Caspase-9.

Firstly, to ascertain the effect of inhibition of CDKs on the viability of human iPS cells, we treated with different concentrations of dinaciclib and found that 6 nM was sufficient to induce significant cell death within 6 hours of treatment, with more than 90% elimination of iPS cells achieved within 24 hrs (Fig. 1a). To test if the observed effect is due to induction of apoptosis, Annexin V-FITC binding was examined using flow cytometry analysis after 6 hours of dinaciclib treatment. We found that the percentage of Annexin V positive cells was significantly higher in the dinaciclib treated group (49.3% ± 10.7%, n = 3) compared to that in DMSO treated cells (10.3% ± 2.9%, n = 3) (Fig. 1b). Additionally, cleaved active Caspase-9 could be detected within 6 hrs of treatment (Fig. 1c).

### Low concentration dinaciclib induced apoptosis in human iPS cells is p53 dependent

**Figure 2:**
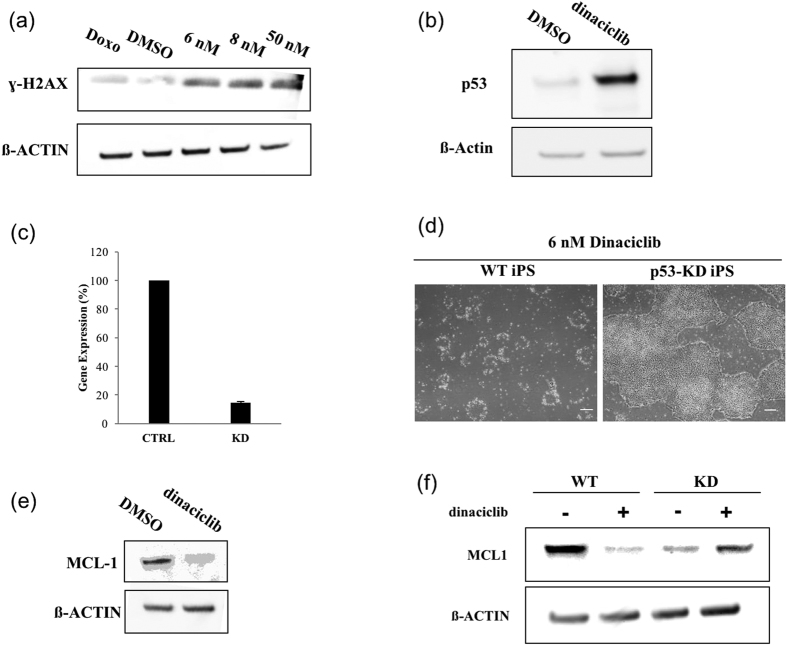
Low concentration dinaciclib induced apoptosis in human iPS cells is p53 dependent. (**a**) iPS cells cultured on laminin E8 fragment were treated with doxorubicin (1 μM), DMSO or different concentrations of dinaciclib for 6 hrs. Whole cell lysates were then used for Western Blot analysis to measure ɣ-H2AX levels. ß-ACTIN was used as a loading control. (**b**) Whole cell lysates from iPS cells treated with DMSO or 6 nM dinaciclib for 6 hrs were used to examine protein levels of p53. (**c**) Total mRNA was extracted from iPS cells transduced with control (CTRL) or p53-specific shRNAs (KD) and was used to measure levels of knockdown using qRT-PCR. (**d**) Representative phase-contrast images of p53 WT and KD iPS cells treated with 6 nM dinaciclib for 24 hrs. Scale bar = 100 μm. (**e**) Western blot analysis of MCL-1 in iPS cells treated with DMSO or 6 nM dinaciclib for 6 hrs. (**f**) Western Blot of MCL-1 in p53 WT and KD iPS cells treated with DMSO or 6 nM dinaciclib for 6 hrs.

Since CDK1 is involved in DNA repair, we aimed to investigate the effect of dinaciclib on the levels of DNA damage in human iPS cells^22^,^23^. To do so, whole cell lysates from cells treated with DMSO or varying dinaciclib concentrations for 6 hrs were examined by Western Blot for ɣ-H2AX signal. We observed that the levels of DNA damage following dinaciclib treatment have increased when compared to the DMSO treated group (Fig. 2a). Since the tumor suppressor and transcription factor p53 is vital for the DNA damage response pathway, we examined its levels in iPS cells following dinaciclib treatment. We found that dinaciclib caused a significant increase in p53 protein levels within 6 hrs of treatment (Fig. 2b). To test whether the observed apoptosis was p53 dependent or not, we utilized a p53 specific shRNA and we were able to generate a stable p53 knockdown iPS cell line with 85% downregulation (±0.9%, n = 3) (Fig. 2c). We found that p53 knockdown inhibited dinaciclib induced apoptosis at 6 nM (Fig. 2d). This suggests that p53 may play a major role in the apoptosis induced by dinaciclib in human iPS cells.

It has been previously reported that chemical inhibition of CDK1 in murine ESC results in p53 mediated increase in *Noxa* mRNA levels and subsequent proteolytic degradation of the anti-apoptotic protein, MCL-1^17^. We have also observed a decrease in MCL-1 protein levels following dinaciclib treatment in human iPS cells (Fig. 2e). Additionally, we found that the decrease in MCL-1 caused by treatment with 6 nM dinaciclib was p53 dependent, as levels of MCL-1 protein were not decreased in the p53-knockdown cells following treatment (Fig. 2f). This finding suggests that, like in mouse ESCs, human iPS cells seem to be sensitive to decreased MCL-1 levels.

### Dinaciclib induces apoptosis in human iPS cells independent of p53 through CDK9 inhibition

**Figure 3:**
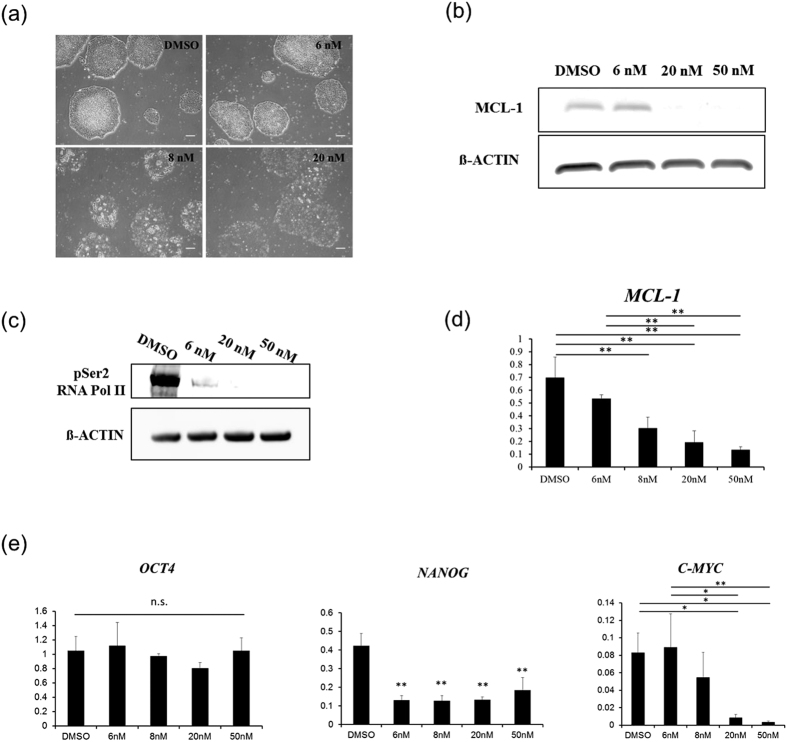
Higher dinaciclib concentration induces apoptosis in iPS cells independent of p53. (**a**) Phase contrast images of p53-KD iPS cells treated with the indicated dinaciclib concentrations for 24 hrs. Scale bar = 100 μm. (**b**) Western blot analysis for MCL-1 protein levels in p53-KD iPS cells treated with the indicated dinaciclib concentrations for 6 hrs. (**c**) WT iPS cells treated with the indicated dinaciclib concentrations for 6 hrs. Whole cell lysates from the treatments were then used to examine the levels of phosphorylated Ser2 of RNA Pol II with Western Blot. (**d**) qRT-PCR showing the levels of MCL-1 mRNA in iPS cells treated with different concentrations of dinaciclib (n = 3) for 6 hrs. Y-axis indicates relative gene expression compared with GAPDH. **p < 0.01 vs. DMSO. (**e**) qRT-PCR showing the levels of mRNAs of indicated genes in iPS cells that were treated with different dinaciclib concentrations for 6 hrs. Y-axis indicates relative gene expression compared with GAPDH. n.s., not significant, *p < 0.05. **p < 0.01.

We have observed that p53-knockdown cells still entered apoptosis when treated with slightly higher dinaciclib concentrations, suggesting the presence of other mechanisms through which dinaciclib may be inducing apoptosis in human iPS cells independently of p53 (Fig. 3a). Interestingly, we have found that treatment of p53-knockdown iPS cell line with higher concentrations of dinaciclib (20 and 50 nM) still caused a significant reduction in MCL-1 protein levels, while MCL-1 levels were maintained in the 6 nM cells (Fig. 3b). Considering the short half-life of MCL-1 and because dinaciclib is known to inhibit the kinase activity of CDK9 and RNA pol II phosphorylation, we hypothesized that the decreased MCL-1 levels caused by treatment with high dinaciclib concentrations might be due to a decrease in the transcription of MCL-1 as a result of a decrease in the number of phosphorylated RNA pol II molecules. As expected we found that treatment of iPS cells with dinaciclib for 6 hrs, caused a rapid dephosphorylation of the CTD serine 2 of RNA Pol II (Fig. 3c). To confirm that reduced MCL-1 levels following dinaciclib treatment was also due to hampered transcription and not only the activation of a proteolytic pathway or changes in translation rates, the levels of *MCL-1* mRNA following dinaciclib treatment were measured by qRT-PCR. Human iPS cells were treated with increasing concentrations of dinaciclib for 6 hrs and then total mRNA was extracted and used for analysis. As expected, *MCL-1* mRNA levels were significantly decreased in a dose dependent manner when iPS cells were treated with 8, 20 and 50 nM of dinaciclib (p < 0.01, n = 3) (Fig. 3d).

Interestingly, we observed that even though 6 hrs treatment of dinaciclib significantly reduced the phosphorylation of Ser2 of RNA Pol II, the treatment did not significantly change the levels of *OCT4* mRNA in iPSCs (n = 3) (Fig. 3e). This may indicate that the C-terminal domain (CTD) phosphorylation at Ser2 of RNA Pol II may not be required for *OCT4* transcription, as previously reported for other genes^24^,^25^. On the other hand, mRNA levels of *NANOG* were significantly decreased by all dinaciclib concentration treatments (p < 0.01 vs. DMSO) (Fig. 3e). Additionally, the mRNA levels of *c-MYC* in dinaciclib treated iPS cells showed a dose dependent downregulation (p < 0.05 6 nM vs. 20 nM and p < 0.01 6 nM vs. 50 nM). Taken together, these findings suggest that dinaciclib treatment has detrimental effects on human iPS cells and may be utilized to purge out contaminating undifferentiated cells in iPS-derived bioengineered tissues.

### Dinaciclib selectively eliminates human iPS cells when co-cultured with normal human cardiac fibroblasts (NHCFs)

**Figure 4:**
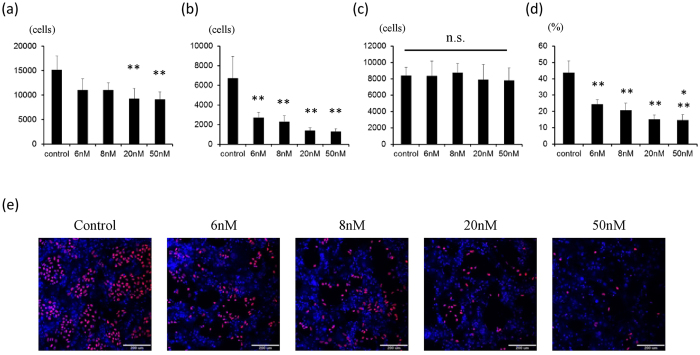
Dinaciclib selectively eliminates human iPS cells when co-cultured with normal human cardiac fibroblasts. (**a**–**c**) The number of cells after dinaciclib treatment. Cell number in 49 fields (7 × 7) was calculated and shown in the graph (n = 4). (**a**) Total cell number. **p < 0.01 vs control. (**b**) Number of OCT4 positive cells. **p < 0.01 vs control. (**c**) Number of OCT4 negative cells. n.s., not significant. (**d**) The percentage of OCT4 positive cells. **p < 0.01 vs control. *p < 0.05 vs 6 nM. (**e**) Representative images of OCT4 positive cells (red). Nuclei were stained with Hoechst (blue). Scale bars = 200 μm.

To test the efficacy of dinaciclib in the selective elimination of iPS cells, we co-cultured human iPS cells with normal human cardiac fibroblasts and then treated with different dinaciclib concentrations. We found that 6 hrs of treatment with all concentrations of dinaciclib resulted in significant reduction in the number of OCT4 positive cells (p < 0.01 vs control) (Fig. 4a,b), while not significantly changing the numbers of OCT4 negative cardiac fibroblasts (Fig. 4c). We could also observe a dose-dependent pattern in the selective elimination of iPS cells with the percentage of OCT4 positive cells being significantly lower in the 50 nM treated wells when compared to the ones treated with 6 nM (p < 0.05) (Fig. 4d). These findings suggest that treatment with 50 nM dinaciclib may be a good strategy to purge out remaining iPS cells in iPS-derived cardiac tissue.

In addition, we have treated non-purified iPS-derived cardiac cells with DMSO or 50 nM dinaciclib for 24 hrs and compared *LIN28* expression using qRT-PCR as an indicator for residual iPS cells. *LIN28* mRNA has recently been shown to be the most sensitive marker for detecting residual iPS cells when co cultured with human primary cardiomyocytes^26^. We have found that dinaciclib treatment decreased *LIN28* to only 46% to what is seen in the control (Supplementary Figure 1). Taken together, these findings suggest that dinaciclib might be suitable to eliminate residual iPS cells in iPS-derived cardiac cells.

### Dinaciclib treatment is not critical for the survival of iPS-derived cardiac cells

**Figure 5:**
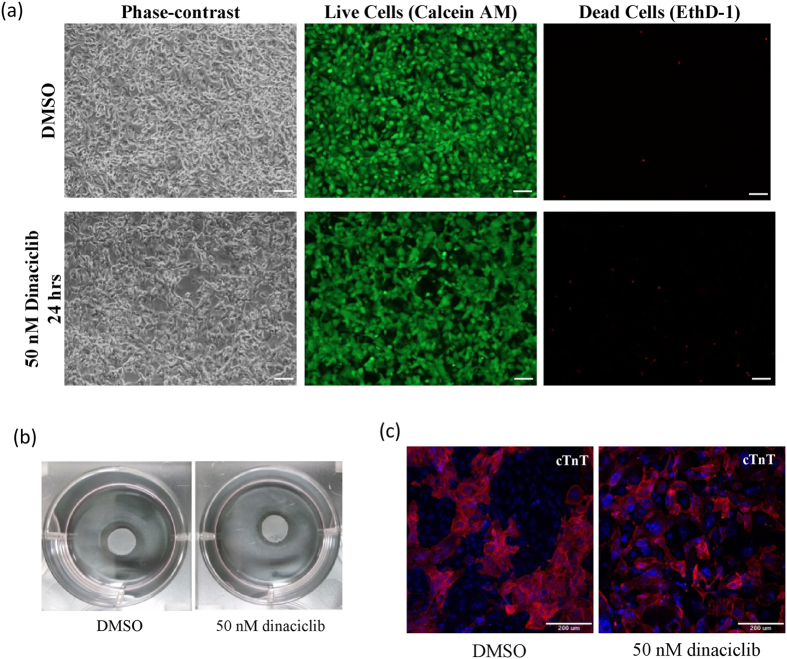
Dinaciclib treatment is not critical for the survival of iPS-derived cardiac cells. (**a**) Purified iPS-derived cardiomyocytes were treated with DMSO or 50 nM dinaciclib for 24 hrs. After 24 hrs of treatment, medium was replaced. The next day cells were stained with Calcein AM (green) and EthD-1 (Red) to detect live and dead cells respectively. Scale bar = 100 μm. (**b**) Non-purified iPS-derived cardiac cells were seeded on temperature responsive dishes pre-coated with FBS. The next day cells were treated with DMSO or 50 nM dinaciclib for 24 hrs. Following treatment, medium was replaced and the next day cell-sheets were obtained by reducing culturing temperature to 20 °C. (**c**) Non-purified iPS-derived cardiac cells were treated with DMSO or 50 nM dinaciclib for 24 hrs. Cells were then fixed in 4% PFA and stained with cTnT (red) and Hoechst (blue). Scale bar = 200 μm.

The efficacy of dinaciclib treatment as a method for residual iPS elimination depends on its influence on iPS-derived bioengineered cardiac tissue. Therefore, after observing that dinaciclib selectively induced apoptosis in human iPS cells, we aimed to investigate its effect on the viability of iPS-derived cardiomyocytes. Human iPSCs expressing puromycin resistance gene under the control of the mouse α-myosin heavy chain (α-MHC) promoter were differentiated towards the cardiac lineage. Since high cell density is required for the fabrication of cardiac cell-sheet, residual iPS cells survival may be enhanced and therefore treatment with higher concentrations of dinaciclib may be needed. Therefore, we treated purified iPS-derived cardiomyocytes with 50 nM dinaciclib for 24 hrs and did not observe considerable cell death (Fig. 5a). Additionally, DMSO and dinaciclib treated cells continued to exhibit spontaneous beating following treatment (Supplementary Video 1 and 2).

Next, we aimed to examine whether or not treatment with dinaciclib might negatively affect the process of cell-sheet fabrication which is necessary for the use of iPS-derived cardiac cells in regenerative medicine. Following differentiation, human iPS cell-derived cardiac cells including cardiomyocytes and fibroblasts were cultured on temperature-responsive culture dishes and transiently treated with DMSO or 50 nM dinaciclib for 24 hrs. Cell sheets were successfully obtained from both treatments after lowering culture temperature (Fig. 5b), which suggests that dinaciclib might not have detrimental effects on any of the necessary elements required for the fabrication of bioengineered tissue, including cell-cell junctions, basement membrane proteins or extracellular matrix. Additionally, when non-purified iPS-derived cardiac cell sheets treated with DMSO or 50 nM dinaciclib for 24 hrs were subcutaneously transplanted in immunodeficient rats, beating could still be seen 7 weeks after transplantation (Supplementary videos 3 and 4). Moreover, when non-purified cardiac cells were treated with 50 nM dinaciclib, the protein expression of cardiac Troponin T (cTnT) was not affected (Fig. 5c). Furthermore, when purified cardiomyocytes were treated with DMSO or 50 nM dinaciclib, the percentage of cTnT positive cell were not affected even after 1 week of treatment (Supplementary Figure 2). Even though, the number of cTnT positive cells was not different 24 hrs following treatment with dinaciclib compared to DMSO, 1 week following treatment, the number of cTnT positive cells was lower in the dinaciclib group compared to DMSO (p < 0.01) (Supplementary Figure 2). Therefore, we speculated that dinaciclib might have some inhibitory effects on the proliferation of cardiomyocytes. As expected, the number and percentage of Ki67 positive cardiomyocytes were significantly (p < 0.05) lower in the 50 nM dinaciclib treated cells (Supplementary Figure 3).

**Figure 6:**
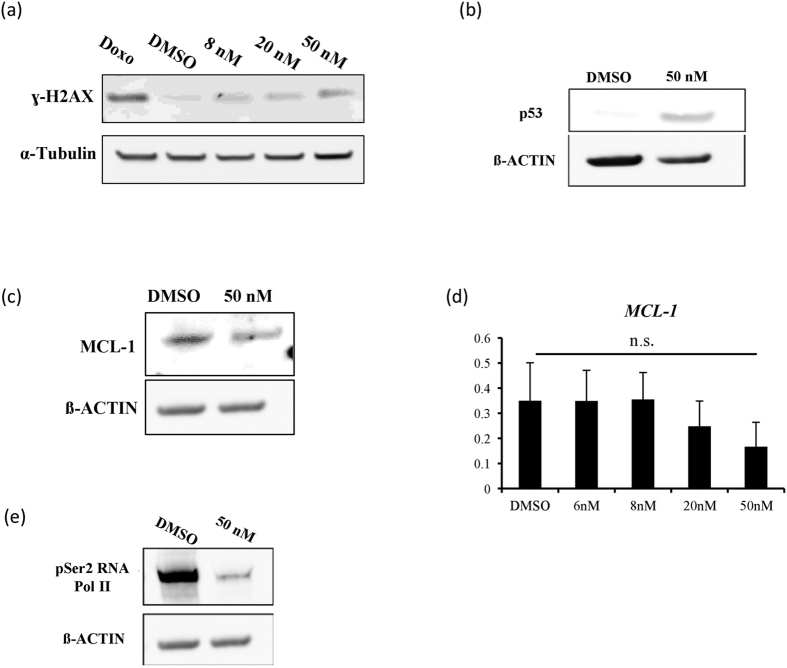
The effect of dinaciclib on iPS-derived cardiac cells. (**a**) Non-purified iPS-derived cardiac cells seeded at the same density used for cell-sheet fabrication were treated for 24 hrs with 1 μM doxorubicin, DMSO or the indicated concentrations of dinaciclib. Two days following the stoppage of treatment, cells were harvested and whole cell lysates were used to examine ɣ-H2AX levels. (**b**, **c**) iPS-derived cardiac cells treated with DMSO or 50 nM dinaciclib and examined for the protein levels of p53 or MCL-1. (**d**) mRNA levels of MCL-1 examined by qRT-PCR in iPS-derived cardiac cells treated with the indicated conditions. Y-axis indicates relative gene expression compared with GAPDH. n.s., not significant. (**e**) Western Blot demonstrating the levels of phosphorylated Ser2 of the CTD of RNA Pol II in iPS-derived cardiac cells treated with DMSO or 50 nM dinaciclib for 24 hrs.

As shown previously, we observed that treatment of iPS cells with dinaciclib increased the levels of ɣ-H2AX. Since causing a permanent increase in DNA damage in iPS-derived cardiac tissue might not be a desirable effect when the tissue is considered for the use in regenerative medicine, we examined the levels of ɣ-H2AX in iPS-derived cardiac tissues that were treated with varying dinaciclib concentrations for 24 hrs, two days after treatment stoppage. We found that levels of ɣ-H2AX in the tissues were only slightly upregulated 48 hrs following the stoppage of treatment (Fig. 6a). We have also observed an increase in the protein levels of p53 after 24 hrs of treatment, which may be due to an initial increase in DNA damage (Fig. 6b). The protein levels of MCL-1 were also slightly decreased 24 hrs following treatment (Fig. 6c). To ascertain whether the decrease of MCL-1 protein is due to proteolytic degradation as a result of p53 stabilization and not due to blockage of transcription, we examined the mRNA levels of MCL-1 by qRT-PCR. We found that 24 hrs treatment of iPS-derived cardiac tissue with dinaciclib did not cause a significant decrease in the transcription of MCL-1 (Fig. 6d). Although there was a significant dephosphorylation of the CTD serine 2 of RNA Pol II (Fig. 6e), some amount of phosphorylated CTD serine 2 of RNA Pol II might be enough to maintain the transcription of *MCL-1* after the treatment with dinaciclib.

## Discussion

In this study, we demonstrate that dinaciclib treatment induces programmed cell death in human iPS cells, but not in their derived cardiac cells. This could allow for the fabrication of a iPS-derived cardiac tissue with a lower risk of contamination of undifferentiated cells. Considering the highly proliferative nature of pluripotent stem cells, the exploitation of the differences in cell cycle regulation between iPS cells and their cardiac derivatives might be a good strategy to purge out remaining iPS cells, especially in iPS-derived cardiac tissue in which cells are not actively dividing.

Dinaciclib, inhibits 50% of the kinase activity of the CDKs 2, 5, 1 and 9 with the concentrations 1, 1, 3 and 4 nM^16^. We found that treatment with 2 nM dinaciclib, which is high enough to inhibit CDK2 and 5 but not CDK 1 or 9, did not induce significant apoptosis in iPS cells (data not shown). This suggests that the inhibition of the CDK2 and 5 might not be detrimental for human iPSCs. This is in line with previous findings that showed that CDK1 can fully compensate for the absence of CDK2^27^,^28^. When we treated human iPS cells with 6 nM dinaciclib, apoptosis was evident in culture within 6 hrs of treatment. More than 90% of the cells became dead within 24 hrs of treatment (Fig. 1a). Previously, it has been shown that specific knockdown of CDK1 in human iPS cells using siRNA did not result in cell death, but caused an increase in differentiation^18^. However, in both cases, using siRNA or dinaciclib to inhibit CDK1 resulted induced an increase in double strand breaks (Fig. 2a)^18^. These findings suggest that dinaciclib treatment at 6 nM is simultaneously inhibiting other CDKs and that the collateral inhibition is necessary for apoptosis to occur in human iPS cells.

We found that dinaciclib treatment caused a significant increase in DNA damage and in p53 protein levels in as early as 6 hrs of treatment (Fig. 2a,b). This was also accompanied by a decrease in the protein levels of the anti-apoptotic protein, MCL-1 (Fig. 2e). Previous reports have shown that when murine and human ESCs were treated with the CDK1 inhibitor, Purvalanol A, a similar response was observed^17^. Furthermore, it was also reported that specific MCL-1 knockdown resulted in cell death in mouse ESCs^17^. Therefore, to confirm whether the observed decrease in MCL-1 following dinaciclib treatment correlated with the occurrence of apoptosis in human iPS cells, and to ascertain if this reduction is caused by proteolytic degradation of the protein mediated by p53 downstream targets (e.g NOXA), we knocked down p53 and treated with dinaciclib. Interestingly, we found that p53 knockdown prevented apoptosis at 6 nM and prevented the observed reduction in MCL-1 protein levels (Fig. 2d,f). These findings suggest that p53 plays a major role in the induced apoptosis following dinaciclib 6 nM treatment in human iPS cells, and that it does so through proteolytic degradation of MCL-1.

Interestingly, we have found that concentrations slightly higher than 6 nM could not be tolerated by p53-knockdown iPS cells (Fig. 3a). Again, the observed apoptosis induced by such concentrations correlated with a decrease in MCL-1 protein levels. This suggested that other mechanisms may come into play and contribute to the reduction of MCL-1 independently of p53. Dinaciclib is also known to inhibit the kinase activity of CDK9 which, within its complex, P-TEFb, functions by phosphorylating Ser2 in the CTD of RNA Pol II, which then promotes the elongation of nascent short transcripts^16^,^20^. We found that only when treated with concentrations higher than 6 nM, a significant reduction in the transcription of *MCL-1* was observed (Fig. 3d). Furthermore, on target inhibition of CDK9 in human iPS cells was confirmed when we examined the levels of phosphorylated RNA Pol II by Western Blot analysis (Fig. 3c).

We have also found that short treatment with dinaciclib induced a significant reduction in the pluripotency genes, *NANOG* and *c-MYC*, while not affecting the transcription of *OCT4* (Fig. 3e). This is in line with a previous study that demonstrated the binding of CDK9 to the regulatory sites of *NANOG* in ESCs^21^. However, it seems that the phosphorylation of the Ser2 of the CTD of RNA Pol II may not be required for *OCT4* transcription. Previous reports have showed that some genes may escape the inhibition of transcription when using pTEFb inhibitors^25^. Therefore, dinaciclib treatment of human iPS cells may concomitantly induce differentiation alongside apoptosis, which are both desired effects when we intend to eliminate residual undifferentiated iPS cells in iPS-derived cardiac tissue.

Since no change in *OCT4* mRNA expression in iPS cells was observed after treatment with dinaciclib (Fig. 3e), *OCT4* transcription may be independent of the Ser2 phosphorylation on the CTD of RNA Pol II. This makes OCT4 a good candidate to detect remaining iPS cells in the co-culture experiment performed in Fig. 4. Therefore, the reduction in OCT4 positive cells in Fig. 4 is highly likely to be a result of selective elimination of iPS cells. Since the data in Fig. 3e represent the relative expression of *OCT4* against *GAPDH*, the decrease of iPS cells in feeder-less condition (iPS cell monoculture) might not affect the relative expression of *OCT4* against *GAPDH.*

We additionally measured *LIN28* mRNA levels using qRT-PCR in non-purified iPS-derived cardiac cells treated with DMSO or 50 nM dinaciclib. *LIN28* mRNA has been shown to be the most sensitive indicator for detecting residual iPS cells when co cultured with human primary cardiomyocytes. This is based on the finding that *LIN28* mRNA could not be detected in primary cardiomyocytes and is highly expressed in human iPS cells when measured by qRT-PCR^26^. Since dinaciclib has been shown to cause a reduction of the phosphorylation of serine 2 of the CTD of RNA Pol II, we speculated that it might reduce the transcription of *LIN28* and falsely exaggerate the dinaciclib mediated elimination of residual iPS cells. Therefore, the medium was replaced after 24 hrs of treatment and cells were harvested for qRT-PCR analysis the day after. We found that *LIN28* expression was decreased to 46% of the levels found in the control (DMSO) (Supplementary Figure 1). Therefore, we believe that dinaciclib treatment might eliminate remaining undifferentiated iPS cells in iPS cell-derived cardiac cells. However, the degree of dinaciclib mediated elimination of residual iPS cells might be difficult to be determined accurately, as it is unclear whether the remaining *LIN28* expression following dinaciclib treatment should be attributed to differences in the degree of maturation between iPS-derived and primary cardiomyocytes or due to persisting undiffereniated cells.

Even though low concentration and short duration dinaciclib treatments were enough to induce DNA damage and apoptosis in iPS cells, purified iPS-derived cardiomyocytes that were 97% positive for cTnT (Supplementary Figure 2) have tolerated dinaciclib with no considerable increase in apoptosis when treated for 24 hrs at 50 nM concentration (Fig. 5a). In addition, 50 nM dinaciclib did not have an effect on the numbers of OCT4 negative NHCF when co-cultured with iPS cells (Fig. 4c). This indicates that cardiac fibroblasts may also have higher tolerance to dinaciclib when compared to iPS cells. Furthermore, dinaciclib treated tissue could be successfully fabricated when iPS-derived cardiac cells (cardiomyocytes and fibroblasts) were cultured on a temperature-responsive dish. Furthermore, the treated cell sheets still exhibited beating 7 weeks after transplantation in nude rats (Supplementary videos 3 and 4). It is worth noting that after 7 weeks, the size of the tissue that was treated with 50 nM dinaciclib prior to transplantation was evidently smaller than the control (DMSO treated). This indicates that the iPS-derived cardiomyocytes utilized in our study possess some degree of proliferative potential that may be reduced with dinaciclib treatment. This is in line with our data presented in (supplementary Figure 3), in which the percentage of Ki67 positive cTnT positive cells were significantly (p < 0.05) lower in the dinaciclib treated cells compared to control. Therefore, there seem to be some inhibitory effects of dinaciclib on iPS-derived cardiomyocytes proliferation.

We have noticed that treatment of iPS-derived cardiac cells with high concentration dinaciclib (50 nM) has induced an increase in p53 protein levels and caused a minute increase in DNA damage. Previous reports have shown that undifferentiated cells may be more prone to undergo apoptosis as a response to DNA damage than differentiated cells^29^. This could be one reason why iPS-derived cardiac cells had higher tolerance to dinaciclib. In non-purified cardiac cells, we observed a modest increase in apoptosis at 24 hrs post the initiation of treatment. This may be due to the presence of undifferentiated or partially differentiated cells that are sensitive to dinaciclib and not due to a loss in the cardiomyocyte constituent of the tissue. This is demonstrated by the persistence of beating of the tissue following treatment (Supplementary video 2)

Treatment of iPS-derived cardiac tissue with dinaciclib has also caused a drastic decrease in the phosphorylation of RNA Pol II (Fig. 6e). However, this decrease in the levels of phosphorylated RNA Pol II did not cause a significant decrease in *MCL-1* transcription (Fig. 6d). This may be due to a higher MCL-1 activity in iPS cells compared to iPS-derived cardiac cells. It has been previously reported that MCL-1 protein levels decrease significantly following the differentiation of murine and human ESCs^17^. Additionally, MYC is known to direct the transcription of MCL-1 and previous reports have shown that tumors that have high MYC activity or are MYC-driven, are specifically sensitive to MCL-1 downregulation compared to MYC independent cancers or normal tissue^30^,^31^,^32^. Therefore, following differentiation of iPS cells to cardiac cells, a reduction in MYC expression is expected, which could possibly lead to a reduction in MCL-1 transcription. Furthermore, CDK9 inhibition by dinaciclib treatment has been shown to induce apoptotic responses in aggressive MYC-driven B-cell lymphoma through transcriptional blockage of *MCL-1*^33^. Taken together, human iPS cells seem to be dependent on the levels of MCL-1, and its indirect inhibition by dinaciclib induces apoptosis. It is also worth mentioning that Western Blot analysis of iPS-derived cardiac tissue did reveal a small decrease in MCL-1 protein level (Fig. 5c). This might be mediated by the increased p53 protein levels in iPS-derived cardiac cells (Fig. 5b) which could be causing proteolytic degradation of MCL-1.

Many groups including us reported the strategies to eliminate remaining iPS cells based on the differences between undifferentiated iPS cells and their derivatives. Based on the evidence that showed high activity of survivin in undifferentiated PSCs compared to their derivatives, Mitsui *et al*. have generated conditionally replicating adenoviruses (CRAs) in which the survivin promoter regulated the adenoviral early region 1 A (E1A) and hence the replication of the virus. Upon treatment with the adenovirus undifferentiated human PSCs were selectively eliminated with no cell death in their differentiated counterparts^34^. Tateno and colleagues have generated a recombinant lectin-toxin fusion protein (rBC2LCN-PE23), made by the fusion of the catalytic domain of *Pseudomonas aeruginosa* exotoxin A, with the lectin rBC2LCN which was previously shown to selectively bind human iPS cells^35^. The negative selection using cell surface markers that specifically express in iPS cells such as CD30 also might be the promising strategy to eliminate iPS cells^36^. The difference in tolerance against methionine free culture condition and TRPV-1 activation between iPS cells and iPS cell-derive cardiac cells has been reported to be capable of eliminating iPS cells^37^,^38^,^39^. It remains unclear whether a single strategy from the ones mentioned above is sufficient to prevent tumor formation in the clinical settings. Combining the use of dinaciclib with other methods might prove more effective in iPS cell elimination. However, the most efficient combination of techniques is yet to be determined.

There are some limitations in this study. We have mainly utilized 30 day-old iPS-derived cardiomyocytes (30 days post-induction of differentiation) to elucidate the effect of dinaciclib on iPS-derived cardiomyocytes. It is possible that the effect of dinaciclib treatment may vary depending on the maturity of iPS-derived cardiomyocytes with immature cells being more sensitive to dinaciclib than older more mature cardiomyocytes. As mentioned above, cardiomyocytes used in this study exhibited some degree of proliferative potential and have increased in number to around 2.5 in one week (Supplementary Figure 2). However, since 50 nM dinaciclib did not induce the cell death of cardiomyocytes (Fig. 5), the toxicity of dinaciclib might not be evident in immature cardiomyocytes. Additionally, when non-purified iPS-derived cardiac cells (23–25 days old), were treated with 50 nM dinaciclib, the beating of the cells was not affected *in vitro* (supplementary video 2) or *in vivo* (supplementary video 4) and the expression of cTnT was not affected (Fig. 5c), indicating that dinaciclib might not have significant cytotoxicity on immature cardiomyocytes. Although dinaciclib decreased the number of iPS cells when co-cultured with human cardiac fibroblasts, it remains unclear to what extent is dinaciclib treatment capable of eliminating the residual iPS cells due to the lack of spike-in experiments.

Results from this study demonstrate that treatment of iPS-derived cardiac tissue with dinaciclib might be an effective method for eliminating residual iPS cells. Dinaciclib treatment of iPS-derived cardiac cells did not affect the process of cell-sheet fabrication. Additionally, beating in the tissue persisted after treatment and the expression of the cardiac marker cTnT was maintained. Dinaciclib is effective in inducing apoptosis in human iPS cells as it targets both CDK1 and CDK9. The inhibition of CDK1 and CDK9 caused the downregulation of the anti-apoptotic protein MCL-1, by proteolytic degradation and by blockage of transcription respectively. Considering the highly proliferative nature of iPS cells compared to their cardiac derivatives, this study demonstrates the feasibility of CDKs inhibition as a method to produce safer cardiac tissue for regenerative medicine.

## Materials and Methods

### Antibodies and reagents

For immunocytochemistry: mouse monoclonal anti-cardiac troponin T antibody (cTnT; Thermo Scientific, Rockford, IL, USA), goat polyclonal anti-OCT4 antibody (R&D systems, Minneapolis, MN, USA), rabbit polyclonal anti-Ki67 antibody (Abcam, Cambridge, UK). FITC, Cy3 or Cy5 conjugated secondary antibodies were used (Jackson ImmunoResearch, West Grove, PA, USA). Nuclei were stained with Hoechst33258 (TheremoFisher, Waltham, MA, USA). For Western blot: rabbit polyclonal anti-actin was obtained from Cell Signaling (Danvers, MA, USA), rabbit polyclonal anti-α-tubulin, rabbit polyclonal anti-ɣ-H2AX and rabbit polyclonal anti-pSer2 RNA Pol II antibodies were obtained from Abcam (Cambridge, UK). Mouse monoclonal anti-p53 and goat polyclonal anti-cleaved caspase-9 antibodies were obtained from Santa Cruz (Santa Cruz, CA, USA) and rabbit polyclonal anti-MCL-1 antibody was obtained from Cell Signaling. Secondary peroxidase-labelled anti-mouse and anti-rabbit were obtained from (GE Healthcare, Marlborough, MA, USA). Secondary peroxidase labeled anti-goat was obtained from Santa Cruz. Dinaciclib (SCH 727965) was obtained from Adoq Bioscience (Irvine, CA, USA).

### Human iPSC culture

Human iPS cell lines (253G1, 201B7) were purchased from RIKEN (Tsukuba, Japan). hiPS cells (201B7) expressing α-MHC promoter and rex-1 promoter-driven drug-resistance genes were established as previously described^39^. 253G1 iPS cells were maintained on iMatrix511 (Nippi, Tokyo, Japan) in StemFit AK03N (Ajinomoto, Tokyo, Japan). Cells were passaged as single cells every 7–8 days using TrypLE Select (Life Technologies, Carlsbad, CA, USA) as described previously^40^. 201B7 iPS cells were maintained on inactivated mouse embryonic fibroblasts (MEFs, ReproCELL, Yokohama, Japan) and were cultured as described previously. Sample images were obtained by an inverted microscope (Nikon, Tokyo, Japan) using the NIS-Elements software (Nikon).

### Cardiac differentiation in the bioreactor and cardiac cell sheet preparation

The cardiac differentiation protocol in the bioreactor system (ABLE Co., Tokyo, Japan) has been described previously^39^. In short, we induced the cardiac differentiation of human iPS cells for 16 days in 3D suspension culture system and then dissociated single cells with trypsin were cultured for 14 days in cell culture plates. In the process of cultivation on cell culture plates, we treated cells with puromycin (Sigma Aldrich) (1.5 μg/ml) twice to purify cardiomyocytes. Therefore, we used 30 day-old cardiomyocytes for experiments. For cell sheet fabrication, when puromycin purification was not performed, the cardiomyocytes were around 23–25 days old. To make the cell sheet, the surface of temperature-responsive dishes (UpCell; CellSeed, Tokyo, Japan) was coated with FBS for 3 hrs prior to seeding. Single cells were plated onto 12 or 24-well plates at 5 × 10^6^ or 1.5 × 10^6^ cells per well respectively in DMEM (Sigma-Aldrich, St. Louis, MO, USA) supplemented with 10% FBS at 37 °C in humid air with 5% CO2. For cell sheet dissociation, the plates were incubated at 20 °C in humid air with 5% CO2.

### Cell sheet transplantation

All animal experiments were performed according to the Guidelines of Tokyo Women’s Medical University on Animal Use, and consistent with the Guide for the Care and Use of Laboratory Animals prepared by the Institute of Laboratory Animal Resources (ILAR). All the experimental protocols were approved by the Institutional Animal Care and Use Committee of Tokyo Women’s Medical University. Two monolayered non-purified iPS cell-derived cardiac cell sheets that were treated with DMSO or 50 nM dinaciclib for 24 hrs were collected by lowering culture temperature and one cardiac cell sheets from each condition was transplanted onto the subcutaneous tissue of a male fischer 344 athymic nude rat (aged around 6 weeks) (Charles River Japan, Tokyo, Japan) as described previously^39^. Seven weeks after transplantation, rats were anaesthetized by 2% isoflurane inhalation and macroscopic images were recorded using surgical microscope system (Leica M651 Surgical microscope system, Germany).

### Lentivirus generation and p53 knockdown in 253G1 feeder-free iPS cells

p53 specific sequence and control shRNAs were obtained from Addgene (Cambridge, MA, USA). Lentiviral particles were produced using the second-generation packaging system obtained from Addgene as reported previously^41^. In short, 10 μg of p53 or control shRNA plasmid, 7.5 μgs of packaging plasmid (psPAX2 plasmid) and 2.5 μg of envelope plasmid (pMD2.G plasmid) were transfected in HEK293t cells using FUGENE 6 (Promega, Fitchburg, WI, USA) transfection reagent according to manufacturer’s instructions. Medium was replaced 24 hrs following transfection. Two days later, lentivirus containing medium was collected and concentrated using Lenti-x concentrator (Takara Bio, Mountain View, CA, USA). Lentivirus particles were re-suspended in OPTI-MEM (Life Technologies), aliquoted and stored in −80 °C for later use. 253G1 human iPS cells were transduced with lentiviral particles using 6 μg/ml polybrene (San Cruz Biotech). When confluent, transduced cells were passaged and expanded. Successfully transduced cells were selected using 2 μg/ml puromycin and were used for experiments.

### RNA extraction and quantitative RT-PCR

Total RNA was extracted using RNeasy kit (QIAGEN, Hilden, Germany). RNA concentration and purity were determined using NanoDrop 2000. Equal amounts of RNA were then used from all samples to construct complementary DNA (cDNA) strand using the High-Capacity cDNA Reverse Transcription Kit (Life Technologies) according to manufacturer instructions. Quantitative PCR was performed with a 7300 Real Time PCR System (Applied Biosystems (ABI), Foster City, CA, USA). Relative mRNA expression levels were calculated using the ΔCT quantification method and endogenous *GAPDH* expression was used for normalization. All primers were obtained from ABI, MCL-1: Hs01050896_m1, POU5F1: Hs00999632_g1, NANOG: Hs02387400_g1 and c-MYC: Hs00153408_m1. LIN28: Hs00702808_s1.

### Protein extraction and Western Blot

TrypLE Select (0.5x) (Life Technologies) and 0.05% trypsin were used to harvest iPS cells and iPS-derived cardiomyocytes respectively. Pelleted cells were treated with 5% protease inhibitor (Cell Signaling) in RIPA buffer (Life Technologies) vortexing occasionally and incubating on ice for 15 mins. Protein concentration was then determined using BCA Protein Assay Kit (Life Technologies). For shearing DNA in cells, protein was sonicated for 5 minutes. Protein extracts were centrifuged at 12,000 rpm at 4 °C for 5 minutes and supernatant was collected for Western Blot analysis. Sodium dodecyl sulfate polyacrylamide gel electrophoresis (SDS-PAGE) and western blotting analysis were carried out as previously described^41^. The membrane was photographed by the chemiluminescence imager (LAS 4000; GE Healthcare).

### Immunocytochemistry

Cells were fixed in 4% paraformaldehyde (PFA) for 12 mins at room temperature and then washed 3 times with PBS and then staining was performed as previously described^35^. Nuclei were stained using Hoechst 33258 (Sigma-Aldrich). Samples were imaged (49 images) by ImageXpress (Molecular Device, Sunnyvale, CA, USA) with MetaXpress and AcuityXpress software (Molecular Device).

### Live/Dead staining

For iPS cells, cells were treated with DMSO or 6 nM dinaciclib and Live/Dead staining was performed at 7 and 24 hrs post treatment using (LIVE/DEAD Viability/Cytotoxicity Kit, Life Technologies) by following manufacturer’s instructions. For purified iPS-derived cardiac tissue, cells were treated with DMSO or 50 nM dinaciclib for 24 hrs. The next day treatments were removed and the cells were stained 24 hrs later. Sample images were obtained by an inverted microscope (Nikon) using the NIS-Elements software (Nikon).

### Annexin-V and flow cytometry analysis

Semi-confluent feeder free 253G1 hiPS cells were treated with DMSO or 6 nM dinaciclib for 6 hrs and then harvested and incubated with Annexin V using the Annexin V-FITC Apoptosis Detection Kit obtained from Abcam according to the manufacturer’s protocol. Cells were then analyzed by flow cytometry using Gallios (Beckman Coulter, Brea, CA, USA).

### Co-culture experiment

NHCF (PromoCell, Heidelberg, Germany) were isolated from adult ventricle tissue and these cells express CD90, but not smooth muscle cell actin or slow muscle myosin, when examined by immunohistochemical staining. On day0, around 1 × 10^5^ NHCF were seeded onto 24-well plates in 10% FBS DMEM. On day1: 1 × 10^5^ cells (feeder-free 253G1 iPS cells) were seeded onto the NHCF. The culture medium was changed from 10%FBS DMEM to StemFit AK03N with Y27632 (10 μM) (Wako, Tokyo, Japan). On day2: The culture medium was changed from StemFit AK03N with Y27632 to 10%FBS DMEM with DMSO or Dinaciclib (6 nM, 8 nM, 20 nM and 50 nM). Six hrs after the treatment, cells were fixed with 4% PFA and subjected to immunostaining.

### Statistical analysis

Data are presented as mean ± standard deviation. Statistical analyses were performed with the Student’s t-test for comparison between two groups. Multiple group comparisons were performed by one-way analysis of variance followed by Tukey-Kramer procedure for comparison of means. A value of p < 0.05 was considered statistically significant.

## Additional Information

**How to cite this article**: Alsayegh, K. *et al*. Dinaciclib potently suppresses MCL-1 and selectively induces the cell death in human iPS cells without affecting the viability of cardiac tissue. *Sci. Rep.* **7**, 45577; doi: 10.1038/srep45577 (2017).

**Publisher's note:** Springer Nature remains neutral with regard to jurisdictional claims in published maps and institutional affiliations.

## Supplementary Material

Supplementary Information

Supplementary Video 1

Supplementary Video 2

Supplementary Video 3

Supplementary Video 4

## References

[b1] Menasche P., Alfieri O., Janssens S., McKenna W., Reichenspurner H., Trinquart L. . The myoblast autologous grafting in ischemic cardiomyopathy (MAGIC) trial: First randomized placebo-controlled study of myoblast transplantation. Circulation 117(9), 1189–1200 (2008). 18285565 10.1161/CIRCULATIONAHA.107.734103

[b2] Bolli R., Chugh A. R., D′Amario D., Loughran J. H., Stoddard M. F., Ikram S. . Cardiac stem cells in patients with ischaemic cardiomyopathy (SCIPIO): Initial results of a randomised phase 1 trial. Lancet (London, England) 378(9806), 1847–1857 (2011). 10.1016/S0140-6736(11)61590-0 PMC3614010 22088800

[b3] Malliaras K., Ibrahim A., Tseliou E., Liu W., Sun B., Middleton R. C. . Stimulation of endogenous cardioblasts by exogenous cell therapy after myocardial infarction. EMBO Molecular Medicine 6(6), 760–777 (2014). 24797668 10.1002/emmm.201303626 PMC4203354

[b4] Zhang M., Methot D., Poppa V., Fujio Y., Walsh K. & Murry C. E. Cardiomyocyte grafting for cardiac repair: Graft cell death and anti-death strategies. Journal of Molecular and Cellular Cardiology, 33(5), 907–921 (2001). 11343414 10.1006/jmcc.2001.1367

[b5] Suzuki K., Murtuza B., Beauchamp J. R., Brand N. J., Barton P. J., Varela-Carver A. . Role of interleukin-1beta in acute inflammation and graft death after cell transplantation to the heart. Circulation 110(11 Suppl 1), II219–24 (2004). 15364866 10.1161/01.CIR.0000138388.55416.06

[b6] Hofmann M., Wollert K. C., Meyer G. P., Menke A., Arseniev L., Hertenstein B. . Monitoring of bone marrow cell homing into the infarcted human myocardium. Circulation 111(17), 2198–2202 (2005). 15851598 10.1161/01.CIR.0000163546.27639.AA

[b7] Nishida K., Yamato M., Hayashida Y., Watanabe K., Yamamoto K., Adachi E. . Corneal reconstruction with tissue-engineered cell sheets composed of autologous oral mucosal epithelium. The New England Journal of Medicine 351(12), 1187–1196 (2004). 15371576 10.1056/NEJMoa040455

[b8] Sawa Y., Miyagawa S., Sakaguchi T., Fujita T., Matsuyama A., Saito A. . Tissue engineered myoblast sheets improved cardiac function sufficiently to discontinue LVAS in a patient with DCM: Report of a case. Surgery Today 42(2), 181–184 (2012). 22200756 10.1007/s00595-011-0106-4

[b9] Ohki T., Yamato M., Ota M., Takagi R., Murakami D., Kondo M. . Prevention of esophageal stricture after endoscopic submucosal dissection using tissue-engineered cell sheets. Gastroenterology 143(3), 582–8.e1-2 (2012). 22561054 10.1053/j.gastro.2012.04.050

[b10] Matsuura K., Honda A., Nagai T., Fukushima N., Iwanaga K., Tokunaga M. . Transplantation of cardiac progenitor cells ameliorates cardiac dysfunction after myocardial infarction in mice. The Journal of Clinical Investigation 119(8), 2204–2217 (2009). 19620770 10.1172/JCI37456 PMC2719947

[b11] Sekine H., Shimizu T., Dobashi I., Matsuura K., Hagiwara N., Takahashi M. . Cardiac cell sheet transplantation improves damaged heart function via superior cell survival in comparison with dissociated cell injection. Tissue Engineering. Part A 17(23-24), 2973–2980 (2011). 21875331 10.1089/ten.tea.2010.0659

[b12] Zweigerdt R., Olmer R., Singh H., Haverich A. & Martin U. Scalable expansion of human pluripotent stem cells in suspension culture. Nature Protocols 6(5), 689–700 (2011). 21527925 10.1038/nprot.2011.318

[b13] Nemunaitis J. J., Small K. A., Kirschmeier P., Zhang D., Zhu Y., Jou Y. M. . A first-in-human, phase 1, dose-escalation study of dinaciclib, a novel cyclin-dependent kinase inhibitor, administered weekly in subjects with advanced malignancies. Journal of Translational Medicine 11, 259-5876–11-259 (2013). 24131779 10.1186/1479-5876-11-259 PMC3853718

[b14] Mita M. M., Joy A. A., Mita A., Sankhala K., Jou Y. M., Zhang D. . Randomized phase II trial of the cyclin-dependent kinase inhibitor dinaciclib (MK-7965) versus capecitabine in patients with advanced breast cancer. Clinical Breast Cancer 14(3), 169–176 (2014). 24393852 10.1016/j.clbc.2013.10.016

[b15] Stephenson J. J., Nemunaitis J., Joy A. A., Martin J. C., Jou Y. M., Zhang D. . Randomized phase 2 study of the cyclin-dependent kinase inhibitor dinaciclib (MK-7965) versus erlotinib in patients with non-small cell lung cancer. Lung Cancer (Amsterdam, Netherlands) 83(2), 219–223 (2014). 10.1016/j.lungcan.2013.11.020 24388167

[b16] Parry D., Guzi T., Shanahan F., Davis N., Prabhavalkar D., Wiswell D. . Dinaciclib (SCH 727965), a novel and potent cyclin-dependent kinase inhibitor. Molecular Cancer Therapeutics 9(8), 2344–2353 (2010). 20663931 10.1158/1535-7163.MCT-10-0324

[b17] Huskey N. E., Guo T., Evason K. J., Momcilovic O., Pardo D., Creasman K. J. . CDK1 inhibition targets the p53-NOXA-MCL1 axis, selectively kills embryonic stem cells, and prevents teratoma formation. Stem Cell Reports 4(3), 374–389 (2015). 25733019 10.1016/j.stemcr.2015.01.019 PMC4375943

[b18] Neganova I., Tilgner K., Buskin A., Paraskevopoulou I., Atkinson S. P., Peberdy D. . CDK1 plays an important role in the maintenance of pluripotency and genomic stability in human pluripotent stem cells. Cell Death & Disease 5, e1508 (2014). 25375373 10.1038/cddis.2014.464 PMC4260724

[b19] Zhou Q., Li T. & Price D. H. RNA polymerase II elongation control. Annual Review of Biochemistry 81, 119–143 (2012). 10.1146/annurev-biochem-052610-095910 PMC4273853 22404626

[b20] Larochelle S., Amat R., Glover-Cutter K., Sanso M., Zhang C., Allen J. J. . Cyclin-dependent kinase control of the initiation-to-elongation switch of RNA polymerase II. Nature Structural & Molecular Biology 19(11), 1108–1115 (2012). 10.1038/nsmb.2399 PMC3746743 23064645

[b21] Wu T., Pinto H. B., Kamikawa Y. F. & Donohoe M. E. The BET family member BRD4 interacts with OCT4 and regulates pluripotency gene expression. Stem Cell Reports 4(3), 390–403 (2015). 25684227 10.1016/j.stemcr.2015.01.012 PMC4375790

[b22] Peterson S. E., Li Y., Chait B. T., Gottesman M. E., Baer R. & Gautier J. Cdk1 uncouples CtIP-dependent resection and Rad51 filament formation during M-phase double-strand break repair. The Journal of Cell Biology 194(5), 705–720 (2011). 21893598 10.1083/jcb.201103103 PMC3171114

[b23] Yu B., Dalton W. B. & Yang V. W. CDK1 regulates mediator of DNA damage checkpoint 1 during mitotic DNA damage. Cancer Research 72(21), 5448–5453 (2012). 22962268 10.1158/0008-5472.CAN-12-2354 PMC3488166

[b24] Gomes N. P., Bjerke G., Llorente B., Szostek S. A., Emerson B. M. & Espinosa J. M. Gene-specific requirement for P-TEFb activity and RNA polymerase II phosphorylation within the p53 transcriptional program. Genes & Development 20(5), 601–612 (2006). 16510875 10.1101/gad.1398206 PMC1410802

[b25] Bensaude O. Inhibiting eukaryotic transcription: Which compound to choose? How to evaluate its activity? Transcription 2(3), 103–108 (2011). 21922053 10.4161/trns.2.3.16172 PMC3173647

[b26] Kuroda T., Yasuda S., Matsuyama S., Tano K., Kusakawa S., Sawa Y. . Highly sensitive droplet digital PCR method for detection of residual undifferentiated cells in cardiomyocytes derived from human pluripotent stem cells. Regenerative Therapy 2, 17–23 (2015). 10.1016/j.reth.2015.08.001 PMC6581767 31245455

[b27] Berthet C., Aleem E., Coppola V., Tessarollo L. & Kaldis P. Cdk2 knockout mice are viable. Current Biology: CB 13(20), 1775–1785 (2003). 14561402 10.1016/j.cub.2003.09.024

[b28] Ortega S., Prieto I., Odajima J., Martin A., Dubus P., Sotillo R. . Cyclin-dependent kinase 2 is essential for meiosis but not for mitotic cell division in mice. Nature Genetics 35(1), 25–31 (2003). 12923533 10.1038/ng1232

[b29] Smith A. J., Nelson N. G., Oommen S., Hartjes K. A., Folmes C. D., Terzic A. . Apoptotic susceptibility to DNA damage of pluripotent stem cells facilitates pharmacologic purging of teratoma risk. Stem Cells Translational Medicine 1(10), 709–718 (2012). 23197662 10.5966/sctm.2012-0066 PMC3659660

[b30] Labisso W. L., Wirth M., Stojanovic N., Stauber R. H., Schnieke A., Schmid R. M. . MYC directs transcription of MCL1 and eIF4E genes to control sensitivity of gastric cancer cells toward HDAC inhibitors. Cell Cycle (Georgetown, Tex.) 11(8), 1593–1602 (2012). 10.4161/cc.20008 22456335

[b31] Kelly G. L., Grabow S., Glaser S. P., Fitzsimmons L., Aubrey B. J., Okamoto T. . Targeting of MCL-1 kills MYC-driven mouse and human lymphomas even when they bear mutations in p53. Genes & Development 28(1), 58–70 (2014). 24395247 10.1101/gad.232009.113 PMC3894413

[b32] Grabow S., Delbridge A. R., Aubrey B. J., Vandenberg C. J. & Strasser A. Loss of a single mcl-1 allele inhibits MYC-driven lymphomagenesis by sensitizing pro-B cells to apoptosis. Cell Reports 14(10), 2337–2347 (2016). 26947081 10.1016/j.celrep.2016.02.039

[b33] Gregory G. P., Hogg S. J., Kats L. M., Vidacs E., Baker A. J., Gilan O. . CDK9 inhibition by dinaciclib potently suppresses mcl-1 to induce durable apoptotic responses in aggressive MYC-driven B-cell lymphoma *in vivo*. Leukemia 29(6), 1437–1441 (2015). 25578475 10.1038/leu.2015.10 PMC4498453

[b34] Mitsui K., Ide K., Takayama A., Wada T., Irie R. & Kosai K. Conditionally replicating adenovirus prevents pluripotent stem cell-derived teratoma by specifically eliminating undifferentiated cells. Molecular Therapy. Methods & Clinical Development 2, 15026 (2015). 26269798 10.1038/mtm.2015.26 PMC4533615

[b35] Tateno H., Onuma Y., Ito Y., Minoshima F., Saito S., Shimizu M. . Elimination of tumorigenic human pluripotent stem cells by a recombinant lectin-toxin fusion protein. Stem Cell Reports 4(5), 811–820 (2015). 25866158 10.1016/j.stemcr.2015.02.016 PMC4437484

[b36] Sougawa N., Masuda S., Miyagawa S., Fukushima S., Ito E., Saito A. . Abstract 14832: Immunologic targeting of CD30 eliminates tumorigenic human pluripotent stem cells (iPSC) allowing safer clinical application of hiPSC-based therapy. Circulation 130(Suppl 2), A14832–A14832 (2014). 10.1038/s41598-018-21923-8 PMC5829260 29487310

[b37] Shiraki N., Shiraki Y., Tsuyama T., Obata F., Miura M., Nagae G. . Methionine metabolism regulates maintenance and differentiation of human pluripotent stem cells. Cell Metabolism 19(5), 780–794 (2014). 24746804 10.1016/j.cmet.2014.03.017

[b38] Matsuura K., Kodama F., Sugiyama K., Shimizu T., Hagiwara N. & Okano T. Elimination of remaining undifferentiated induced pluripotent stem cells in the process of human cardiac cell sheet fabrication using a methionine-free culture condition. Tissue Engineering. Part C, Methods 21(3), 330–338 (2015). 25245976 10.1089/ten.TEC.2014.0198

[b39] Matsuura K., Seta H., Haraguchi Y., Alsayegh K., Sekine H., Shimizu T. . TRPV-1-mediated elimination of residual iPS cells in bioengineered cardiac cell sheet tissues. Scientific Reports 6, 21747 (2016). 26888607 10.1038/srep21747 PMC4757885

[b40] Nakagawa M., Taniguchi Y., Senda S., Takizawa N., Ichisaka T., Asano K. . A novel efficient feeder-free culture system for the derivation of human induced pluripotent stem cells. Scientific Reports 4, 3594 (2014). 24399248 10.1038/srep03594 PMC3884228

[b41] Alsayegh K. N., Gadepalli V. S., Iyer S. & Rao R. R. Knockdown of CDK2AP1 in primary human fibroblasts induces p53 dependent senescence. PloS One 10(3), e0120782 (2015). 25785833 10.1371/journal.pone.0120782 PMC4365013

