# Supplementary material for: Dinaciclib potently suppresses MCL-1 and selectively induces the cell death in human iPS cells without affecting the viability of cardiac tissue
Source: Sci Rep. 2017 Mar 31;7:45577. doi: 10.1038/srep45577 (PMC5374522; doi:10.1038/srep45577)
Supplement: Supplementary Information [file srep45577-s1.pdf]

Dinaciclib potently suppresses MCL-1 and selectively induces cell death in human iPS cells without affecting the viability of cardiac tissue

Khaled Alsayegh[1,2], Katsuhisa Matsuura[1,3*], Hidekazu Sekine[1], Tatsuya Shimizu[1].

Supplementary figures:

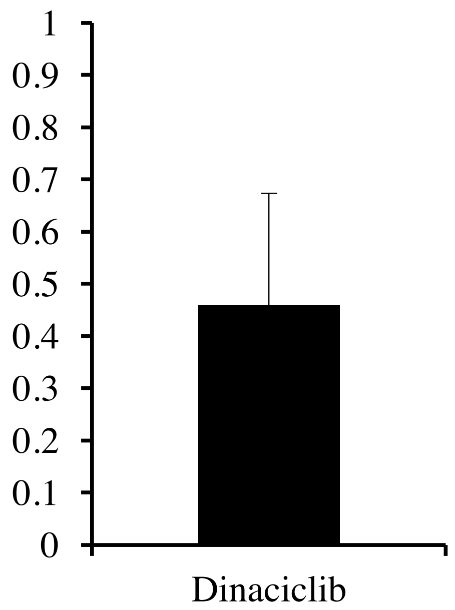

Supplementary figure 1: Dinaciclib treatment reduced *LIN28* expression in iPS-derived cardiac cells. Human iPS-derived cardiac cells were treated with DMSO or 50 nM dinaciclib for 24 hrs. Following treatment, the medium was replaced and the next day the cells were harvested for qRT-PCR analysis. The graph demonstrates a reduction in the *LIN28* mRNA to around 46% compared to DMSO (n=6).

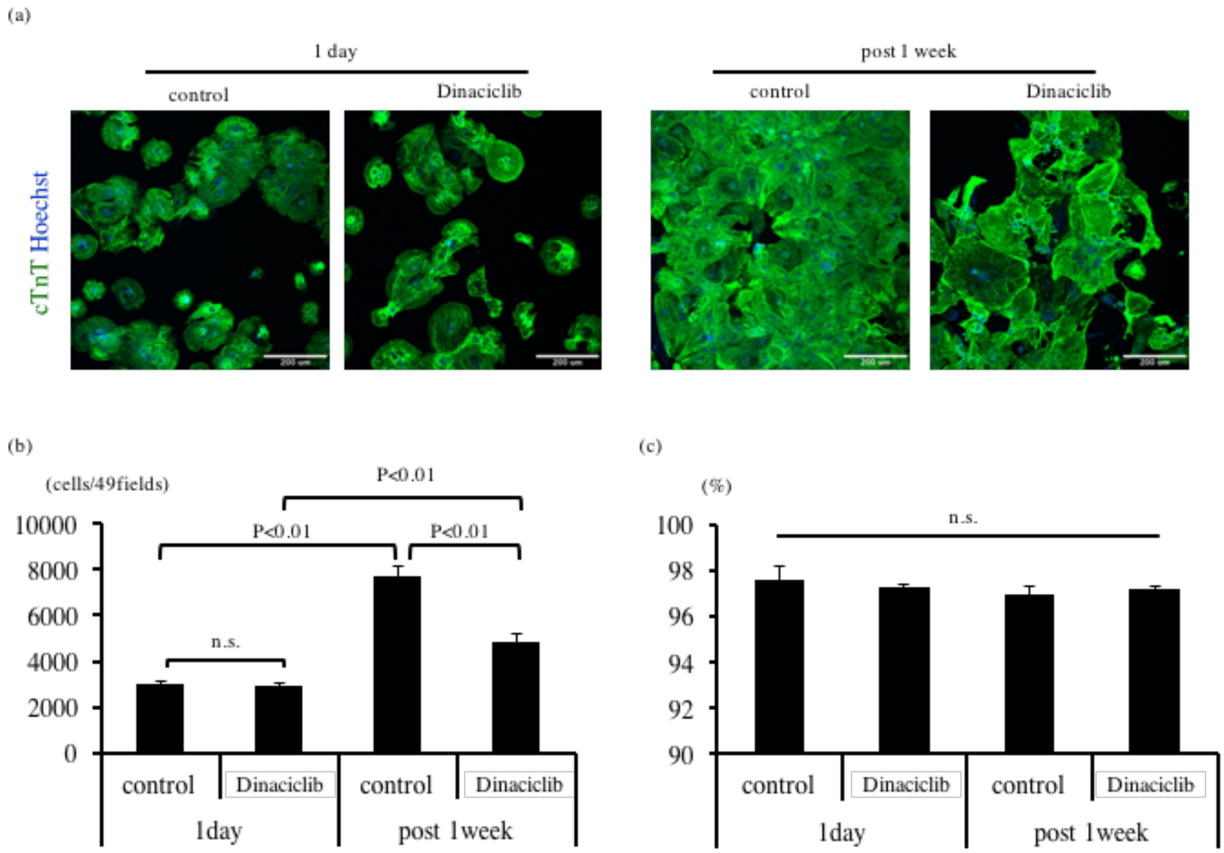

Supplementary figure 2: Long term effect of dinaciclib on human iPS-derived cardiomyocytes. (a-c) Purified cardiomyocytes were cultured with DMSO as control or dinaciclib (50nM) for 1 day and then cultured for further 1 week without DMSO or dinaciclib. (a) Representative images of cTnT (green) and Hoechst (blue) staining. Bar, 200 μm. (b, c) The number (b) and percentage (c) of cTnT (+) cells in 49 fields at each time point were calculated and shown in the graph (n=3). n.s., not significant.

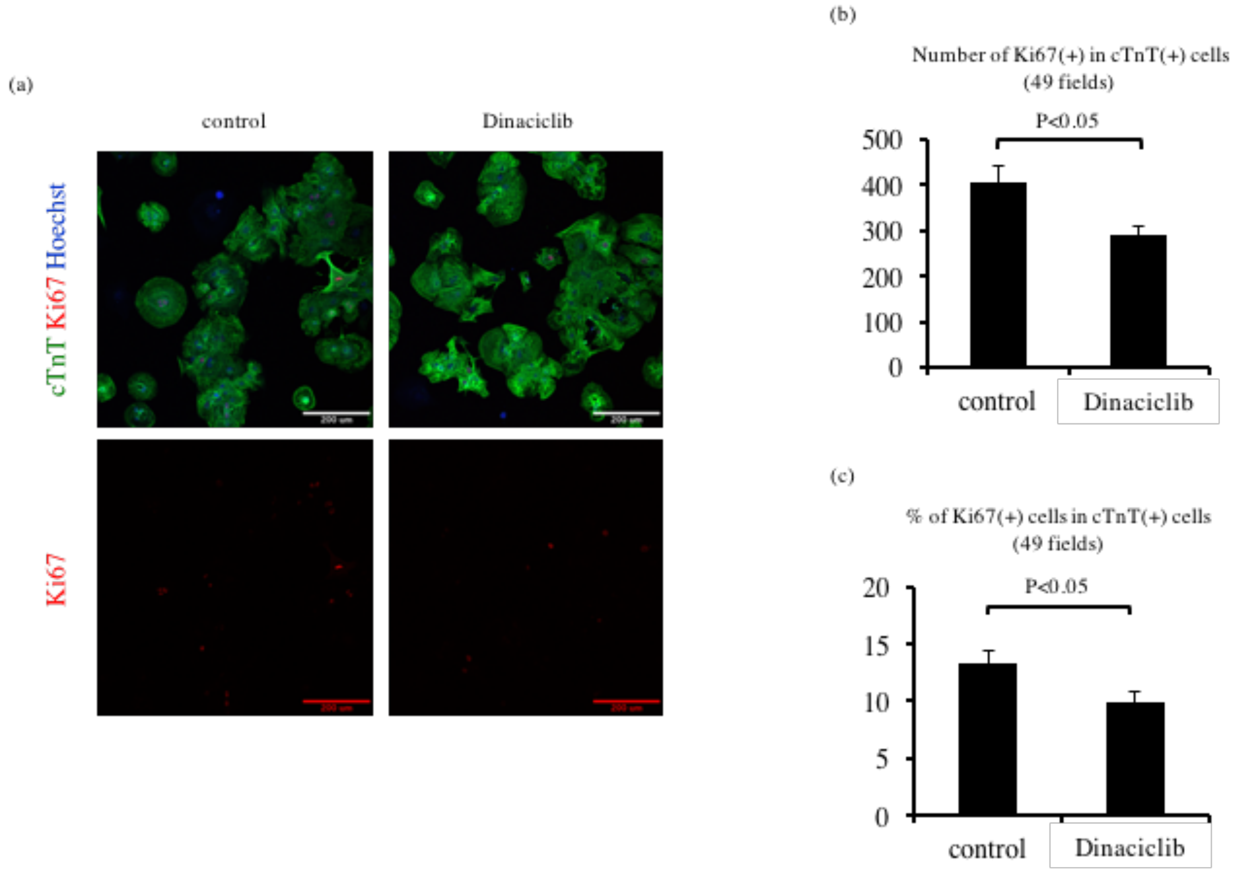

Supplementary figure 3: The effect of dinaciclib on proliferation of iPS-derived cardiomyocytes. (a-c) Purified cardiomyocytes were cultured with DMSO as control or dinaciclib (50nM) for 1 day. (a)Representative images of cTnT (green), Ki67 (red) and Hoechst (blue). Bar, 200 µm. (b-c) The number (b) and percentage (c) of Ki67(+) cells in cTnT(+) cells in 49 fields were calculated and shown in the graph (n=3).

Supplementary video legends.

Supplementary Video 1. Spontaneous beating of iPS cell-derived cardiomyocytes treated with DMSO for 24 hrs. The original magnification is ×10.

Supplementary Video 2. Spontaneous beating of iPS cell-derived cardiomyocytes treated with 50 nM dinaciclib for 24 hrs. The original magnification is ×10.

Supplementary Video 3. Asynchronous beating of iPS cell-derived cardiac cell sheet treated with DMSO for 24 hrs and subcutaneously transplanted in nude rats for 7 weeks.

Supplementary Video 4. Asynchronous beating of iPS cell-derived cardiac cell sheet treated with 50 nM dinaciclib for 24 hrs and subcutaneously transplanted in nude rats for 7 weeks.
